# Anthropometric measurements as an indicator of nutritional status in spina bifida patients undergoing enterocystoplasty

**DOI:** 10.1590/S1679-45082013000200006

**Published:** 2013

**Authors:** Silvia Ferraz Ayrosa Ponte, Atila Rondon, Herick Bacelar, Eulalio Damazio, Sandra Maria Lima Ribeiro, Gilmar Garrone, Valdemar Ortiz, Antonio Macedo

**Affiliations:** 1 Universidade Federal de São Paulo, São Paulo, SP, Brazil

**Keywords:** Meningomyelocele, Urinary bladder, neurogenic, Nutritional evaluation, Child

## Abstract

**Objective:**

To use anthropometric measurements to compare nutritional status in children with neurogenic bladder dysfunction secondary to meningomyelocele who underwent enterocystoplasty and those who did not undergo surgery.

**Methods:**

A case-control study was conducted in 20 children, divided into two groups: those who had enterocystoplasty (Group A) and those who did not undergo surgery (Group B), matched for genre and age. Weight, height, arm circumference, and triceps skinfold thickness were the parameters used. Nutritional assessment was determined by calculating the indexes, based on age and genre. Classification was based on the percentile and the results were compared with the reference values.

**Results:**

The mean age was 6.41 years in Group A and 6.35 years in Group B. The interval between surgery and evaluation was 11 months. The following measures were found for Group A: 80% of children were eutrophic, a percentage 30% greater than that in Group B; arm muscle circumference was adequate in 40% of patients, a percentage 20% greater than that in Group B; arm muscle area was adequate in 90%, a percentage 30% greater than that in Group B. Values in Group B were as follows: for triceps skinfold thickness, 60% of patients had values above the mean, a percentage 20% greater than that in Group A; for arm fat index, 60% of patients were above the mean value, 40% greater than in Group A.

**Conclusion:**

Patients who had undergone enterocystoplasty showed better nutritional status, while the control group presented higher fat indexes in anthropometric measures. However, the differences between groups were not statistically significant.

## RESUMO

**Objetivo:**

Utilizar as medidas antropométricas para comparar o estado nutricional em crianças com bexiga neurogênica secundária à mielomeningocele submetidas a enterocistoplastia àquelas não submetidas à cirurgia.

**Métodos:**

Estudo tipo caso-controle conduzido em 20 crianças, divididas em dois grupos, aquelas que realizaram enterocistoplastia (Grupo A) *versus* crianças não operadas (Grupo B), pareados por gênero e idade. Os parâmetros utilizados foram peso, altura, circunferência do braço e prega cutânea triciptal. A avaliação nutricional foi determinada calculando-se índices baseados na idade e no gênero. A classificação foi baseada em porcentagens, e os resultados foram comparados com valores de referência.

**Resultados:**

A média da idade foi 6,41 anos no Grupo A e de 6,35 no Grupo B. O intervalo entre a cirurgia e a avaliação foi de 11 meses. As seguintes medidas foram encontradas para o Grupo A: 80% das crianças eram eutróficas, 30% maior do que no Grupo B; circunferência braquial foi adequada em 40% dos pacientes, 20% maior do que no Grupo B; a área do músculo do braço foi adequada em 90%, 30% maior do que no Grupo B. Os valores no Grupo B foram os seguintes: 60% apresentaram prega cutânea triciptal acima do valor médio, uma porcentagem 20% acima do que no Grupo A; para índice de gordura braquial, 60% dos pacientes estavam acima da média, 40% maior do que no Grupo A.

**Conclusão:**

Pacientes submetidos à enterocistoplastia demonstraram melhor estado nutricional enquanto o grupo controle apresentou índices de gordura mais elevados nas medidas antropométricas. Entretanto, a diferença entre os grupos não foi estatisticamente significativa.

## INTRODUCTION

Myelomeningocele (MMC) occurs when the neural tube fails to close. The incidence of this neurologic abnormality in Brazil is 2.28 per 1,000 live births^(1)^. In these patients, 50% of all postnatal problems concern the urogenital system, and 40% of these will require a urologic procedure to reduce injury to the upper urinary tract, avoid urinary tract infections, or promote urinary and fecal continence^(2,3)^. Bowel segments are frequently used to replace the bladder, but this approach can lead to metabolic disorders^(2,3)^ and compromise the patient's nutritional status. Wagstaff et al. identified growth deficits in 20% of children in whom colon segments were used^(4)^. The authors subsequently reviewed the same sample, taking into account underlying diseases, age, and surgery; this analysis indicated that 15% of patients presented accelerated growth^(5)^. In a prospective study of 15 patients, Clementson Kockum et al. found no change in growth as a result of surgery^(6)^.

Children with MMC are a heterogeneous group with different complex chronic disorders^(7)^. They are at high risk for nutritional disorders, such as insufficient growth profile, obesity, osteoporosis, diabetes, and cardiovascular diseases. Nutritional care analyses are essential to estimate growth curves, changes in weight, and speed of growth in order to define preventive or curative interventions to minimize complications. The anthropometric method is efficient, simple, and fast as well as considered the best approach in this area^(8–10)^. However, we faced challenges in performing anthropometric testing because of the orthopedic characteristics of patients with MMC^(8,10–13)^.

## OBJECTIVE

The goal of this study was to use anthropometric data to evaluate the nutritional status of children with neurogenic bladder due to myelomeningocele, matched for genre and age, who did or did not undergo enterocystoplasty.

## METHODS

This cross-sectional, case-control study was conducted among 20 children with neurogenic bladder (NB) due to MMC. The children were 3 to 11 years of age and had not yet undergone puberty. We initially selected from our database ten children who had had enterocystoplasty and paired them with ten controls according to genre and age. The controls had not undergone enterocystoplasty. Thus, the two study groups were Group A (Augmentation; n=10) and Group B (Control; n=10).

The anthropometric evaluation in Group A was performed within 6 months after the enterocystoplasty. At the initial consultation, patients were measured for weight, estimated height, arm circumference, and triceps cutaneous fold.

Weight was calibrated by using an anthropometric digital scale, with a W-200A balance (Welmy) with capacity of 200kg (±50g). Patients remained erect in the center of the scale, the arms parallel to the body, wearing undergarments only (underpants for girls and briefs for boys) and without shoes. Patients who could not stand upright without support were weighed in the lap of their caregiver, and the caregiver's weight was subsequently discounted^(14)^.

The height was obtained by estimation via measurement of the right upper arm length because many patients could not walk. An inextensible metric tape with millimeter scale was used to measure the distance from the acromion to the top of the radius with the upper limb flexed to 90°^(14–17)^. Estimated height was calculated from the equation proposed by Stevenson: estimated height in centimeters = (4.35 × upper arm length) 21.8±1.7 standard deviation^(17)^.

The mid-upper arm circumference was measured with the right arm extended at the midpoint between the acromion process and the olecranon, using an inextensible metric tape with millimeter scale^(14–16)^.

The triceps skinfold thickness was lifted by a Lange adipometer (±1mm), above the midpoint of the arm's length. The measurement was made for three consecutive times and the mean value was calculated^(14–16)^.

From these measurements the following indexes were determined: height in relation to age, arm circumference relative to age, triceps skin fold in relation to age, muscular arm circumference relative to age, arm muscle area in relation to age, arm fat area in relation to age, and arm fat index age. For classification purposes, the growth scale provided by the World Health Organization was used as standard reference; this scale was also adopted by the National System of Nutritional Surveillance (www.saude.gov.br/nutricao) in 2007^(18)^. We organized our data and presented the Z score as an additional measure to assess nutritional status.

To classify the ability to walk, we considered the following possibilities: capable of walking: remains standing without support; partially capable: needs support or orthoses; unable to walk: needs to use a wheelchair.

The survey was registered with and approved by the Committee of Ethics and Research CEPE (registration number: 5440). After we identified potential candidates for the study, the adults who were responsible for the children were sent an invitation letter and a consent form that clarified the scope and purpose of the research. The research protocol was initiated after the informed consent document was signed.

For statistical evaluation, descriptive analyses were carried out in the form of means, medium and maximum values, and standard deviations, along with scatter plot diagrams. Qualitative variables were analyzed by calculating absolute and relative frequency and plotted bar charts. The Fisher exact test or its extension was used to test the association between groups, and the Student t test was used for comparison. The significance level was set at 5%.

## RESULTS

The sample was composed of 20 patients, divided into Group A (Augmentation) and Group B (Control). Each group consisted of nine girls and one boy, who were paired up by genre and age. The mean age of children in the two groups was similar. The mean height and weight of children in both groups did not significantly differ (Table 1).

**Table 1:** Age, height and weight on the day of the anthropometric evaluation

Variables	Samples		Total	p value*
	Group A (Augmentation)	Group B (Control)		
Age (years)	6.41 (3.16-10.83)	6.35 (3.16-10.05)	6.38 (3.16-10.83)	0.947
Weight (kg)	17.77 (11.3-26.6)	17.72 (13-27)	17.75 (11.3-27)	0.980
Estimated height (cm)	113.15 (100-135)	114.68 (104.45-126)	113.91 (100-135)	0.692

* p value according to Student t test for independent samples.

Children in Group A were subjected to nutritional evaluation after a 6-month minimum follow-up (mean: 11 months; range: 6 to 31 months).

In Group A, 50% of the children were able to walk, while in Group B none were able to walk.

Both groups showed statistically equal profiles regarding nutritional measures. However, some indexes showed a tendency toward better nutritional conditions. For example, for body mass index (BMI) in relation to age, 80% of the children in Group A were eutrophic (suitable), a percentage 30% greater than that in Group B. For triceps skinfold thickness in relation to age, 60% of children in Group B had values above the mean, a percentage 20% greater than that in Group A. Arm muscle circumference in relation to age was adequate in 40% of the children in Group A, which was 20% greater than the percentage in Group B. Arm muscle area in relation to age was adequate in 90%, a percentage 20% greater than that in Group B. In Group B, 60% of children were above mean value for arm muscle area, compared with 20% in Group A.

Table 2 classifies nutritional status of the children in accordance with all anthropometric variables evaluated. Tables 3 and 4 show data on nutritional status expressed according to percentile of BMI and Z score for BMI among study groups.

**Table 2:** Classification of the nutritional status of the children in accordance with all anthropometric variables evaluated

		Samples		Total	p value*	Power
		Group A n (%) (Augmentation)	Group B n (%) (Control)			
H/A	Below the mean	1 (10)	2 (20)	3 (15)	>0.999	0.096
	Adequate	9 (90)	8 (80)	17 (85)		
BMI/A	Below the mean	2 (20)	4 (40)	6 (30)	>0.350	0.336
	Eutrophic	8 (80)	5 (50)	13 (65)		
	Above the mean	–	1 (10)	1 (5)		
AC/A	Below the mean	2 (20)	3 (30)	5 (25)	>0.999	0.084
	Eutrophic	6 (60)	5 (50)	11 (55)		
	Above the mean	2 (20)	2 (20)	4 (20)		
TSF/A	Below the mean	2 (20)	–	2 (10)	0.554	0.341
	Eutrophic	4 (40)	4 (40)	8 (40)		
	Above the mean	4 (40)	6 (60)	10 (50)		
AMC/A	Below the mean	6 (60)	7 (70)	13 (65)	0.628	0.262
	Eutrophic	4 (40)	2 (20)	6 (30)		
	Above the mean	–	1 (10)	1 (5)		
AMA/A	Below the mean	10 (10)	2 (20)	3 (15)	0.582	0.242
	Eutrophic	9 (90)	7 (70)	16 (80)		
	Above the mean	–	1 (10)	1 (5)		
AFA/A	Below the mean	4 (40)	1 (10)	5 (5)	0.146	0.510
	Eutrophic	4 (40)	3 (30)	7 (35)		
	Above the mean	2 (20)	6 (60)	8 (40)		
AFI/A	Below the mean	2 (20)	2 (20)	4 (20)	>0.999	0.080
	Eutrophic	4 (40)	3 (30)	7 (35)		
	Above the mean	4 (40)	5 (50)	9 (45)		

* p value according to exact Fisher's test or its extension.

H/A: height for age; BMI/A: body mass index for age; AC/A: arm circumference for age; TSF/A: triceps skin fold for age; AMC/A: arm muscle circumference for age; AMA/A: arm muscle area for age; AFA/A: arm fat area for age; AFI/A: arm fat index for age.

**Table 3:** Nutritional status according to body mass index, percentile and Z score among study groups

Group A - Augmentation			
BMI	Percentile/Age	Z Score/Age	Post-operative status
15.2	P50	0	Eutrophic
17.3	P85	1	Eutrophic
14.8	P15	1	Eutrophic
13.7	P15	1	Eutrophic
15.9	P50-P85	Between 0 and 1	Eutrophic
13.3	P3-P15	Between −2 and + 1	Eutrophic
13.6	↑P3	Between −2 and + 1	Eutrophic
13.0	P3-P15	Between −2 and +1	Eutrophic
8.8	↓P3	−3	Thinness
12.1	↓P3	−3	Thinness
**Group B - Control**			
**BMI**	**Percentile/Age**	**Z Score/Age**	**Pre-operative status**
13.2	P3-P15	Between −2 and + 1	Eutrophic
14.1	P15	Between −1 and 0	Eutrophic
14.48	P15 50	Between −1 and 0	Eutrophic
17.08	P50-P85	Between 0 and 1	Eutrophic
15.6	P50	0	Eutrophic
17.9	↑P85	Between 1 and 2	Overweight
11	↓P3	−3	Thinness
11.3	↓P3	−3	Thinness
11.0	↓P3	−3	Thinness
12.3	↓P3	−3	Thinness

BMI: body mass index.

**Table 4:** Distribution according to Z score classification system

BMI/Age					
< Z score −3	≥ Z score −3 and < Z score −2	≥ Z score −2 and ≤ Z score +1	≥ Z score +1 and ≤ Z score +2	≥ Z score +2 and ≤ Z score +3	> Z score +3
Extreme thinness	Thinness	Eutrophic	Overweight	Obesity	Extreme obesity
**Group A - Augmentation**					
0	2	8	0	0	0
**Group B - Control**					
0	4	5	1	0	0

BMI: body mass index.

## DISCUSSION

This research was conducted to fill a gap in knowledge of the nutritional status of children with NB due to MMC^(3,13,19,20)^. The study compared the nutritional status of children with NB due to MMC who did or did not have bladder augmentation surgery. Results showed a tendency toward better nutritional conditions in patients who underwent enterocystoplasty.

Kurzrock et al. found that when enterocystoplasty is performed during infancy, the patient's recovery and adaptation are better. At adolescence, these children are continent, more independent, and enjoy a better quality of life^(21)^. Donovan et al. studied 60 patients with a mean age at enterocystoplasty of 14 years (range: 6 to 29 years; an age range broader than that of our study) and found complications in 52%^(13)^. The children in Kurzrock's study who underwent this procedure did so primarily at preschool and school ages^(21)^. In contrast, the mean age of our patients was 5.5 years, according to the pre-established criteria; this factor hindered patient selection and limited the size of the comparison group^(13)^.

Littlewood et al. assessed the walking ability of 19 children and adolescents with MMC, grouping them according to the level of the injury. The patients were considered ambulants when injury severity was low (52.6), mean ambulation was considered when the value was mean (15.7), and non-ambulants when injury severity was high (31.57). In our study, no children in Group B could walk, compared with half the children in Group A. These differences were statistically significant (p=0.004), and this finding justified the use of the estimated height from the measure of upper arm length^(22)^. Given our results and those in the study of Littlewood et al., future research should consider physiotherapy evaluation and bone densitometry in order to elucidate these findings.

During this study, children in Group A underwent nutritional evaluation after a minimum follow-up of 6 months, with mean time of 11 months (range: 6 to 31 months). The initial selection of patients who had surgery before the onset of puberty took place over a 3-year period before data analysis; patients were selected from a tertiary center in the public health system affiliated with our university. The social and economic status of patients seen in this health system is similar, thus minimizing variations in nutrition.

According to Donovan et al., the mean follow-up duration was 39 months (range: 13 to 101 months). These authors evaluated the BMI of 60 patients with NB due to MMC who underwent the same surgery and related it to potential complications. The number of postoperative complications in obese patients was twice that in patients who were not obese. From these findings, Donovan et al. suggested that the nutritional status of patients with MMC should always be monitored, especially before surgery, because nutritional interventions and physical activity are recommended to keep patients eutrophic. Doing so, we could minimize complications after surgery^(13)^. This information is relevant, and future studies should consider using a longer follow-up period and additional anthropometric measures to better interpret this variable.

In our study, patients who had surgery did not have a higher rate of complications due to nutritional parameters compared with the Control Group, but this could be due to the small number of patients evaluated.

Mingin et al. also found no change in growth after enterocystoplasty in children with NB due to MMC. They compared the growth of 22 children with and without surgery, paired according to genre, age, and lumbar-sacral injury. Three measures of weight and height were collected in the pre- and postoperative periods, and the follow-up time was 3.6 years longer than that of our study. Mingin et al. found that all patients were in the 10^th^ percentile for height and showed no significant change in BMI because growth was not affected in the 59% of patients who underwent the procedure^(3)^.

In a prospective study of 123 children who had enterocystoplasty, Gerharz et al. found that 85% maintained adequate growth and that only four had pathologic growth; this was due not to the surgery but to endocrinopathy^(23)^. Mingin et al., in another study, noted that there was no change in the final height of adults and that patients did not have bone demineralization. In our patients, both groups showed proper height according to standard references^(24)^.

In terms of BMI, 80% of patients in Group A were eutrophic and 20% were below the mean value; no patients were above the mean. In comparison, 50% of patients in Group B were eutrophic, 40% were below the mean value, and 10% were above. The results reported here differ from those obtained by Donovan et al. in 60 patients who had undergone enterocystoplasty; 50% of those patients were eutrophic, 17% were overweight, and 33% were obese^(13)^. This difference between the studies can be attributed to differences in the sample size.

In a controlled study, Grogan et al. evaluated body composition in children with MMC by using potassium levels (K 40), weight, height, waist circumference, and skinfold thickness; they found that waist circumference is related to the percentage of fat, indicating that fat in children with MMC accumulates around the hip. The fat in the lower areas of the body below the neurologic injury was also significantly correlated with fat content, from the total of skinfold thickness. Thus, the use of waist circumference and measures of skinfold thickness to determine fatness in children with MMC is highly recommend and should be initiated as soon as possible because they are easily determined^(9)^. In our study, Group B had the highest percentage of children with adipose content above the mean value, a finding similar to that in Grogan et al.

Group A had more patients with muscle mass above the mean; lean mass above the mean value does not present any health risk. This finding may be associated with the duration of follow-up and sample size.

The comparative analysis of anthropometric indexes among Groups A and B did not allow us to define statistically significant differences that could be interpreted as influenced by the surgery. Note, however, the greater tendencies toward better muscle mass in Group A, expressed by arm muscle circumference and arm muscle area, and larger adipose mass in Group B, expressed by skinfold thickness in relation to age and arm fat index in relation to age.

This study has limitations with regard to sample size. The relatively short follow-up duration may also have been a limiting factor in the results obtained. On the other hand, the study does represent a homogeneous sample in prepubertal phase, and to our knowledge there are few reports like this in the literature. We know of no other prospective studies evaluating nutritional status in the MMC population undergoing enterocystoplasty. We acknowledge that despite the cross-sectional case-control nature of the study, the motor status of both groups varied: five patients in Group B relied on wheelchairs compared with none in Group A.

Another difficulty with the study of anthropometric indexes in patients with MMC is the lack of validated standard references for a population with so many variables. As a result, we had to use, and accept as the only means, the World Health Organization growth scale for reference.

Another limitation of our study was that we did not include food consumption analysis, biochemical data, or imaging. If these measures had been included, they would have strengthened the interpretation of the data. On the other hand, social and economic status of families of both groups was similar.

Another similar study is underway, in which nutritional assessment, analysis, and intervention of each patient are being done prospectively, before and after enterocystoplasty; this will enable us to assess whether the surgery and any improvement in the patient's medical condition affect nutritional status. Each child will be compared with himself or herself; doing so we will respect each child's individual characteristics and allow each to become his or her own control.

## CONCLUSIONS

Despite the findings suggesting better nutritional condition in the enterocystoplasty group, the study did not identify statistically significant differences in nutritional status of patients with neurogenic bladder due to MMC, according to whether the patient had undergone surgery or not.
